# Experimental and Numerical Investigation of the Use of Ultrasonic Waves to Assist Laser Welding

**DOI:** 10.3390/ma17112521

**Published:** 2024-05-23

**Authors:** Mohamad Salimi, Ahmed Teyeb, Evelyne El Masri, Samiul Hoque, Phil Carr, Wamadeva Balachandran, Tat-Hean Gan

**Affiliations:** 1 Brunel Innovation Centre, Brunel University London, Uxbridge UB8 3PH, UK; mohamad.salimi@brunel.ac.uk (M.S.); evelyne.elmasri@brunel.ac.uk (E.E.M.); wamadeva.balachandran@brunel.ac.uk (W.B.); 2 Carr’s Welding Technologies Ltd. (CWT), Kettering NN16 8PX, UK; samiul@carrswelding.co.uk (S.H.); pc@carrswelding.co.uk (P.C.); 3 TWI Ltd., Granta Park, Cambridge CB21 6AL, UK

**Keywords:** ultrasound cavitation, laser welding, microscopy, battery cell connectors

## Abstract

This study evaluates the enhancement of laser welding using ultrasonic waves aimed at reorganising the intermetallic position in such a fashion that leads to increased mechanical properties of welds in battery pack assemblies for electric vehicles. The experiment employed 20 kHz and 40 kHz High-Power Ultrasound Transducers (HPUTs) in both contact and contactless modes. A simplified experimental configuration is suggested to represent conditions similar to those found in electric vehicle battery pack assemblies. Measurements of vibration transmission to aluminium alloy 1050 plates revealed more than a 1000-fold increase in acceleration amplitude in contact mode compared to contactless mode. The 20 kHz transducer in contactless mode demonstrated superior performance, showing a 10% increase in load and 27% increase in extension compared to welding without ultrasonic assistance. On the other hand, the 40 kHz transducer, while still improved over non-ultrasonic methods, showed less pronounced benefits. This suggests that lower-frequency ultrasonic assistance (20 kHz) is more effective in this specific context. The study explores ultrasonic assistance in laser welding copper (Cu101) to aluminium alloy 1050 using 20 kHz and 40 kHz HPUTs, showing that both transducers enhance microstructural integrity by reducing copper homogenisation into aluminium, with the 20 kHz frequency proving more effective in this context. A numerical simulation was conducted to evaluate the transmission of pressure into the molten pool of the weld, correlated with the vibration results obtained from the 20 kHz transducer. The numerical simulation confirms that no cavitation is initiated in the molten pool area, and all improvements are solely due to the ultrasonic waves.

## 1. Introduction

Laser welding has emerged as a promising technique for joining components due to its precision, speed, and ability to create strong and durable welds [1]. The strength and quality of laser-welded joints can be enhanced by applying ultrasonic energy to the molten pool. The impact of vibrations on the solidification process of molten metal in casting or fusion welding has been extensively studied and researched throughout the twentieth century [2,3,4].

The effect of ultrasonic vibration on the solidification phase significantly impacts the final structure, mechanical properties, and electrical characteristics of the material [5]. This is particularly critical when dealing with metal alloys or combinations of dissimilar materials, as observed in lap joining for battery cell connectors in the production of electric vehicle battery packs [6,7]. The coexistence of liquid and solid phases, owing to the varying melting points of different metals, leads to the agglomeration of similar particles during solidification, which ideally should be uniformly dispersed throughout the liquid phase [8]. However, commonly observed phenomena such as voids, gas presence, and variable grain sizes contribute to the degradation of the final material’s properties. This degradation gives rise to issues like hot cracking, resulting in fragile battery connections and high electric impedance, causing energy loss and temperature elevation in battery packs [9,10,11]. The occurrence of acoustic cavitation in the molten pool aids in the fragmentation and shaping of intermetallic compounds, which are the primary culprits behind property degradation.

Wong et al. [12] developed pulsing cylindrically converging acoustic waves to induce ultrasound cavitation. Khavari et al. [13] showed the mechanism behind cavitation-driven shock waves, vital for optimising the effectiveness of ultrasound in diverse applications such as sonochemistry and material processing. Usadi et al. [14] developed a machine learning algorithm to monitor and classify cavitation noise in various setups, enhancing its application in biomedicine, sonochemistry, and waste degradation. Preso et al. [15] showed the effect of vapor compression and energy dissipation during the collapse of cavitation bubbles, highlighting the resistance provided by higher vapor pressures to bubble collapse and the subsequent reduction in energy dissipation. Sieber et al. [16] present a boundary integral method-based numerical solver designed for simulating single cavitation bubble dynamics near fluid–fluid interfaces, facilitating detailed studies on cavitation bubble behaviour under various boundary conditions. Preso et al. [17] examine the influence of non-condensable gases on the dynamics and luminescence of cavitation bubbles in water, finding minor effects of air saturation on bubble behaviour. Sieber et al. [18] present a novel method for constructing high-speed movies of collapsing cavitation bubbles from a single long-exposure image using a consumer-level camera, enhancing the study of bubble dynamics with high temporal and spatial resolution.

Subroto et al. [19] explored the impacts of ultrasonic cavitation melt treatment (UST) on the temperature distribution, sump profile, and resulting microstructure during the direct-chill (DC) casting of an AA6008 aluminium alloy. Utilising experimental setups with and without UST, alongside numerical modelling, the research demonstrates that UST alters the temperature gradients and sump profile, leading to a refined grain structure within the billets. Theoretical aspects of using remote ultrasound vibration to initiate cavitation bubbles in the molten pool area during welding are studied by Teyeb et al. [20]. Through computational simulations, the study identifies optimal positions and frequencies for ultrasound vibration to achieve maximum pressure in the fusion zone, thereby enhancing the welding process. The research demonstrates that side excitation of the plate generates significantly higher vibration displacement amplitudes compared to corner excitation, leading to more effective cavitation and improved welding quality. Their results present a comprehensive theoretical framework for understanding the interaction between ultrasound vibration and cavitation dynamics in welding, offering valuable insights for developing more efficient and high-quality welding techniques.

Liu et al. [21] presented a comprehensive investigation into enhancing the efficiency of ultrasonic vibration-assisted laser welding through the optimisation of an ultrasonic oscillator’s design and eigenfrequency tuning. The optimisation aims at mitigating the effects of high-temperature-induced eigenfrequency drift in ultrasonic systems, a common challenge during ultrasonic-assisted laser welding due to the heat transfer from the welding process. The study establishes a dynamic frequency model for the ultrasonic oscillator, integrating a finite element genetic algorithm and temperature field coupling to predict and adjust for eigenfrequency shifts effectively. Zhou et al. [22] innovatively introduce a follow-up ultrasonic-vibration-assisted welding process to address common defects in laser welding of dissimilar metals, specifically between Hastelloy C-276 and stainless steel 304, critical for nuclear reactor pump-can end sealing. The process demonstrates significant improvement in the microstructure and mechanical properties of the weld joint, contributing to the optimised application of ultrasonic vibration. Lei et al. [23] address the challenges of welding porosity and poor weld formability in laser welding processes of magnesium alloys, specifically the AZ31B magnesium alloy. By employing an experimental approach to ultrasonic-assisted laser welding, the research delineates significant enhancements in welding characteristics, including weld defect reduction and microstructure improvements. The introduction of ultrasonic vibration to the weld pool results in a notable decline in weld porosity from 4.3% to 0.9%, alongside a reduction in the average size of the porosities. Dhara and Das [24] investigated the ultrasonic welding process for multi-layered aluminium to copper joints for electric vehicle battery applications, highlighting the significant impact of ultrasonic process parameters on the quality of welds, which was analysed through layer-wise microstructural studies.

The focus of this paper is centred on the use of contactless ultrasonic vibration assistance to improve welding quality. This type of remote vibration is sometimes required when contact vibration is not feasible. For instance, in battery pack assemblies for electric vehicles, it is crucial to avoid subjecting the battery to high-power ultrasonic vibrations that may cause damage to its internal components. The investigation in this study begins with a description of the current battery holder for laser welding and a simplified experimental configuration to present the battery pack assemblies for electric vehicles in Section 2. In Section 3, ultrasonic-assisted laser welding is conducted on aluminium (Al1050) plates, followed by an analysis of the welds through pull tests. Section 4 replicates the same experimental study using aluminium (Al1050) and copper (Cu101) plates, followed by conducting pull tests and microscopy examinations. In Section 5, numerical simulations using COMSOL Multiphysics are performed to elucidate some of the experimental findings and to confirm the initiation of ultrasound cavitation. Finally, conclusions are drawn in Section 6.

## 2. Experimental Configuration for Ultrasonic Assistance Laser Welding

**Figure 1:**
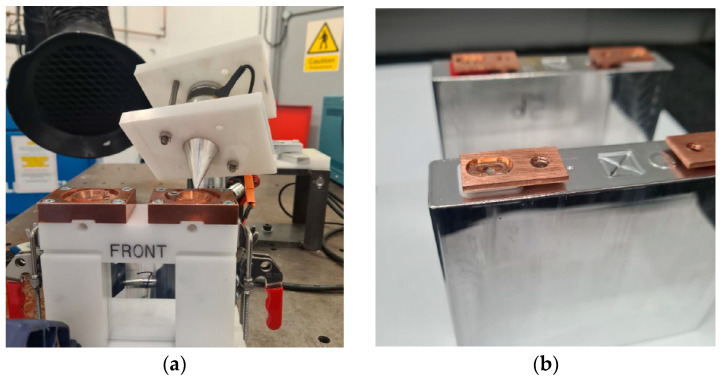
Laser welding of a copper plate to a battery terminal. (**a**) The ultrasonic assistance laser welding configuration. (**b**) The final plate welded to the battery.

In the welding process, a battery is enclosed within a white plastic cover, as depicted in Figure 1a. A small copper (Cu101) plate, as shown in Figure 1b, is then welded to one of the battery terminals.

**Figure 2:**
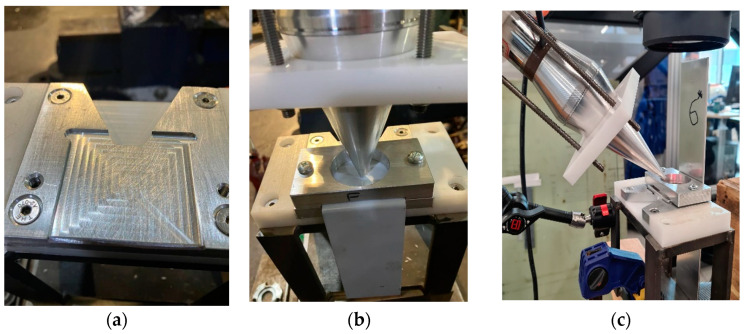
The simplified welding configuration. (**a**) The bottom holder, (**b**) fastened top holder to the bottom one, and (**c**) a picture showing the location of two plates, one at the top and the other at the bottom, along with the location of excitation from the transducer.

The current battery holder has limited space to record the vibration transmission to the little plate terminal, and even using a laser vibrometer, the data were erratic. Furthermore, by welding the plate to the battery head, it is difficult to analyse the welding in terms of the pull test and microscopy. Therefore, a simplified setup is considered to replicate the welding of the plates to the battery terminal, as illustrated in Figure 2.

**Figure 3:**
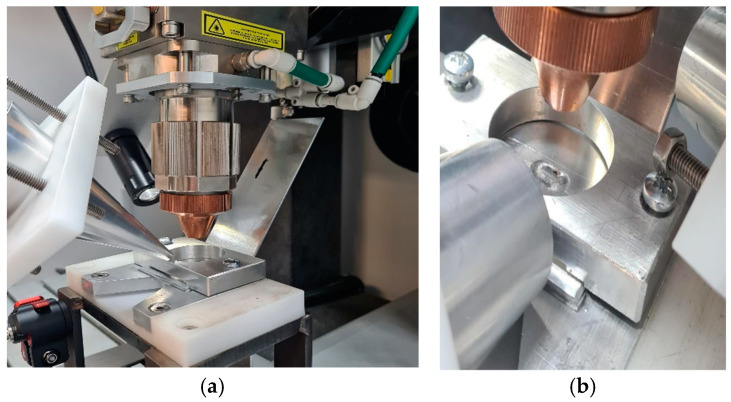
The welding configuration includes (**a**) the utilisation of an ultrasound transducer with a focussed horn during laser welding and (**b**) the oval shape of the welding.

As illustrated in Figure 2b,c, a holder fastens two L-shaped plates, one at the top and the other at the bottom. This simplification allows for the recording of the transmitted vibration in the area where welding will take place. The utilisation of contactless vibration could present a viable solution for ultrasonic-assisted laser welding, particularly when encountering restricted space, as illustrated in Figure 3. 

As seen in Figure 3, the final laser weld of the plate has an oval shape. The lasers used in this study are IPG Photonics high-power multimode Ytterbium Fibre Laser systems and their country of origin is the USA. The laser power utilised was 2000 W. The laser head operated at a speed of 2750 mm/min, with a wobble width of 0.7 mm and a frequency of 250 Hz. All the plates used in this study have an L shape with dimensions of 50 × 50 mm at the welded bottom part and 100 × 50 mm at the top section. Throughout the trials, certain parameters were kept constant. This included the thickness of the plates being welded, which was 0.9 mm for copper and 2 mm for aluminium. All the measurements in this study were conducted with High-Power Ultrasound Transducers (HPUTs) at 20 kHz and 40 kHz, operating at 70% power. It is not recommended to increase the power amplification of the industrial horn as it tends to overheat and produce low amplitude noise instead of the desired signal. The 20 kHz transducer features a horn length of 127 mm, with the large tip having a diameter of 33 mm and the small tip a diameter of 3.3 mm. The 40 kHz transducer has a length of 62 mm, with the large tip having a diameter of 22.5 mm and the small tip a diameter of 2.25 mm.

## 3. Ultrasonic Assistance of Laser Welding

This section investigates the use of contact and contactless vibration methods to enhance laser welding quality by employing 20 kHz and 40 kHz HPUTs with small and large end tip horns, as illustrated in Figure 2 and Figure 3.

Salimi et al. [25] characterised an industrial HPUT to determine the optimal distance and angle for contactless excitation. To make the vibration level at its optimum value, the horn length should be proportional to half of the wavelength associated with the transducer. To mitigate potential heating of the piezoelectric component in a similar transducer used in this study and the amplifier setup during continuous operation, a frequency sweep can be used [26]. This technique indirectly protects the transducer and power generator from potential damage. By varying the operating conditions through a frequency sweep, excessive heat accumulation is prevented, thus preserving the integrity of both the transducer and the power generator. 

### 3.1. Vibration Measurements

**Figure 4:**
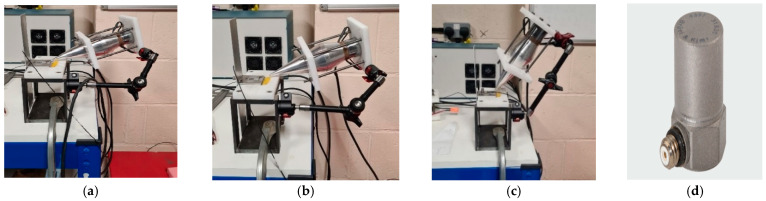
The experimental configuration generates (**a**) contact and (**b**) contactless vibration at approximately 0 degrees and (**c**) contactless vibration at a 45-degree angle. (**d**) High-frequency accelerometer type 4397.

Prior to conducting ultrasonic-assisted laser welding, vibration tests are carried out to determine the vibration levels of the plates under welding in both contact and contactless excitation modes. The experimental vibration measurements involve a 20 kHz HPUT with a small horn end tip. The contact and contactless vibration output from the HPUT were measured on the plates, made of aluminium alloy 1050, as shown in Figure 4a–c. To find the vibration level associated with each measurement, a high-frequency accelerometer type 4397 is used, see Figure 4d, to evaluate plate response.

**Figure 5:**
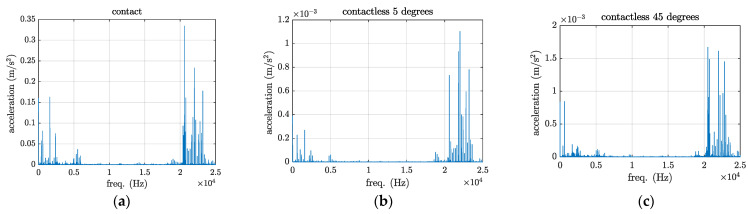
The vibration output from the (**a**) contact, (**b**) contactless excitation, and (**c**) contactless excitation at a 45-degree angle.

The accelerometer is operating on a single axis; hence, to record the plate vibration for the in-plane and out-of-plane excitation, the accelerometer should be oriented in the desired direction. It is expected that the vibration at a zero-degree angle generates an in-plane motion to the plate in the contact and the contactless excitation modes. From previous assessments using the same configuration with a 20 kHz HPUT [25], it was found that maximum vibration occurs when the transducer is positioned at a 60-degree angle, 20 mm away from the end tip of the horn and the plate. Although the 60-degree angle is optimal, a 45-degree angle has been chosen because using the 60-degree angle would result in a clash between the laser machine head and the HPUT. As illustrated in Figure 5, the vibration level from the contact excitation is more than 1000 times higher in acceleration amplitude compared to the contactless excitation. 

### 3.2. Pull Test Results Using 20 kHz Transducer

**Figure 6:**
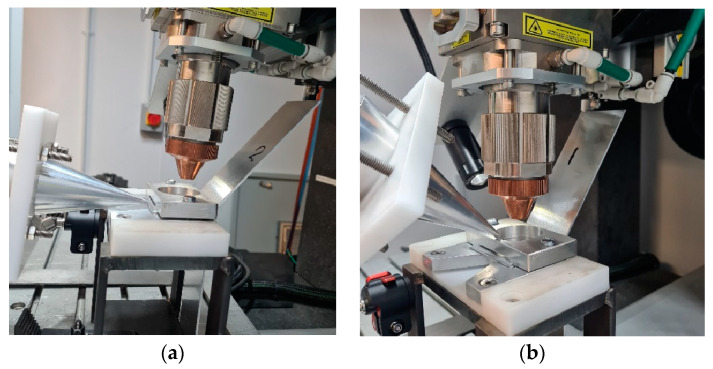
Ultrasonic-vibration-assisted welding using a HPUT with a small end tip horn at (**a**) a 0-degree angle in contact, and (**b**) a 45-degree angle in contactless mode.

For this section, an HPUT with a focused ultrasound horn operating at 20 kHz is used. Both contact and contactless modes are employed to transmit ultrasonic waves into the molten pool area. Figure 6 illustrates the different HPUT positions at a 0-degree angle in contact mode, and at a 45-degree angle in contactless mode. The vibration for contactless excitation was performed with a 20 mm distance from the molten pool area.

**Figure 7:**
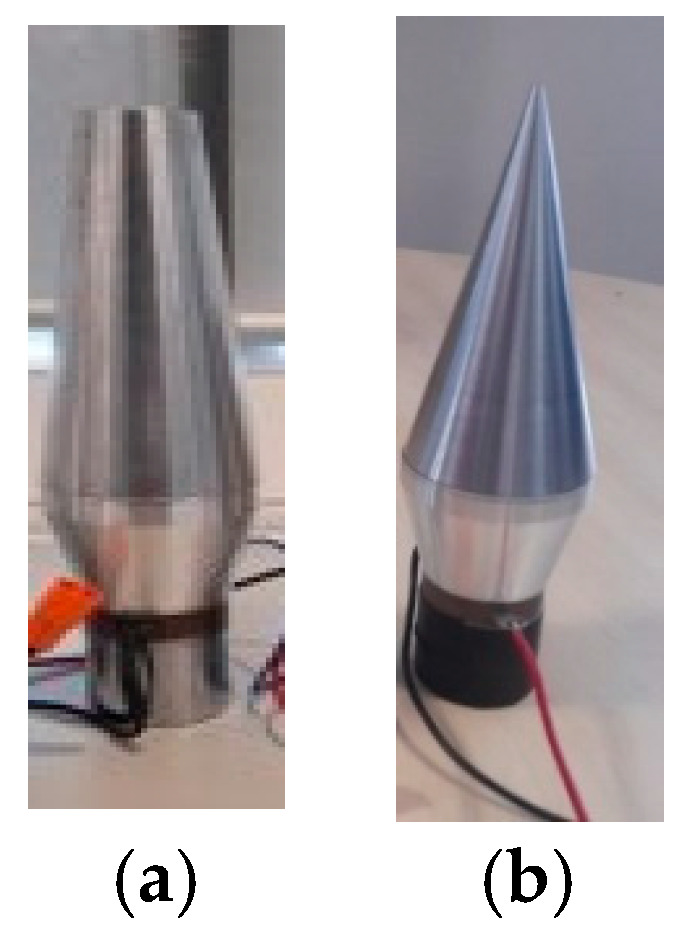
The 20 kHz HPUT used during the laser welding. Transducer configurations: (**a**) a large tip horn and (**b**) a small tip horn.

The 20 kHz HPUT used in this study is depicted in Figure 7. The HPUT, featuring a larger horn tip as shown in Figure 7a, transmits greater overall pressure waves to the molten pool area. Conversely, the HPUT with a smaller horn tip, illustrated in Figure 7b, offers enhanced usability in the constrained spaces typically encountered during welding.

**Figure 8:**
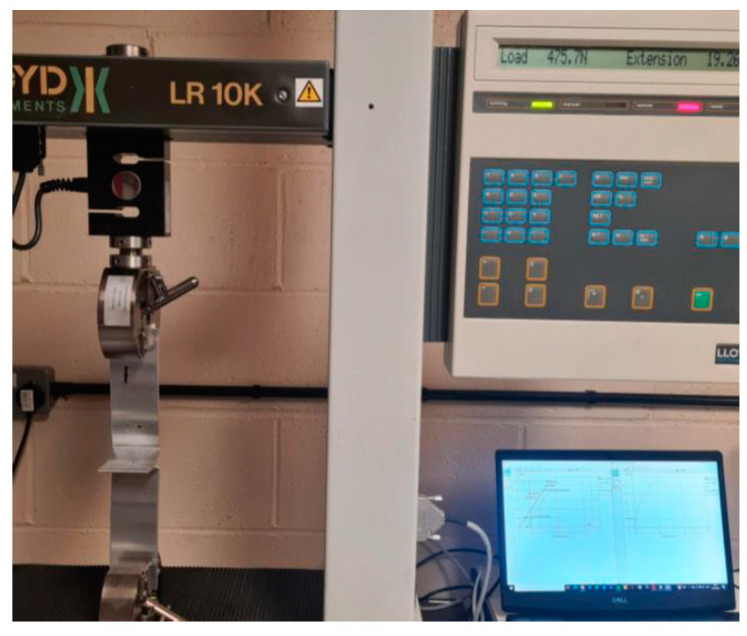
A Lloyd LR10K tensile tester used to conduct pull tests.

The pull tests were conducted based on ISO 4136:2022 [27], ISO 6892-1:2020 [28] and ISO 9018:2015 [29] standards. Tensile tests were performed at room temperature, which is set at 20 °C with a 50% relative humidity. A Lloyd LR10K tensile tester, manufactured by Lloyd Instruments Ltd. at their head office in West Sussex, UK, illustrated in Figure 8, was used to perform these tests, ensuring consistent and reliable measurement in accordance with the specified international standards.

The results from pull tests are summarised in Table 1. A noticeable improvement in contactless mode indicates that low-intensity ultrasonic vibration is more effective in enhancing the mechanical properties of the weld than contact vibration.

The use of the HPUT with the small horn tip in the contactless position demonstrated approximately a 10% increase in load and a 27% increase in extension compared to the non-ultrasonic case. When comparing contactless and contact setups, the HPUT with the small horn tip in the contactless configuration exhibited about a 6% higher load and a 31% greater extension than the large horn tip in a contact configuration. The use of the HPUT with the large horn tip in the contactless position resulted in roughly a 1% increase in load and a 23% increase in extension over the contact position.

### 3.3. Pull Test Results Using 40 kHz Transducer

**Figure 9:**
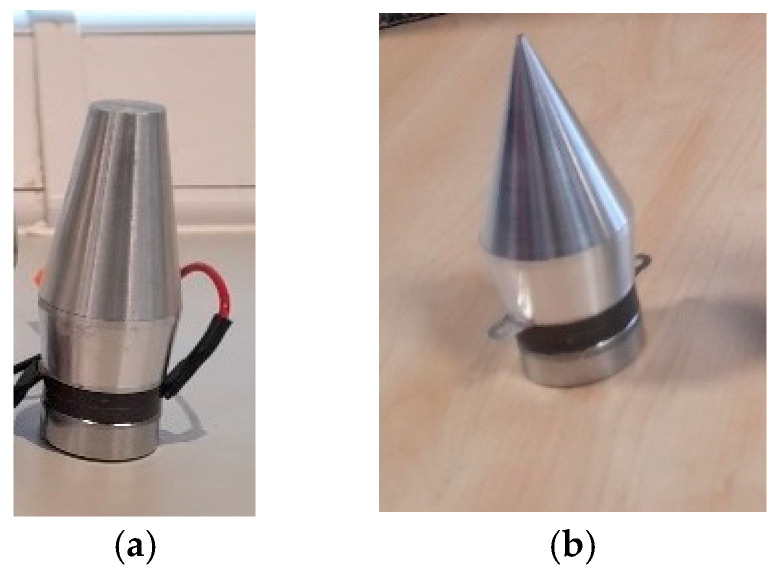
The 40 kHz HPUT used during the laser welding. Transducer configurations: (**a**) large horn tip and (**b**) small horn tip.

In this section, the welding process was carried out using a 40 kHz HPUT. The transducer, characterised by a large and a small horn tip, is illustrated in Figure 9a,b respectively.

The outcomes of the pull tests, corresponding to the use of the 40 kHz transducer in both contact and contactless modes, are depicted in Table 2. The same standard pull test procedure used for assessing the results, as explained in Section 3.2, is considered in this section. Better results in terms of welding strength were obtained compared to those achieved without ultrasonic assistance.

The HPUT with the small tip horn in contactless mode at 40 kHz showed approximately a 4% increase in load and a 16% increase in extension compared to the non-ultrasonic method. The contactless versus contact at 40 kHz with the large tip showed approximately a 1% increase in load and a 34% increase in extension. Comparing 40 kHz to 20 kHz, the best performance with 40 kHz (small tip contactless) fell short by approximately 6.4% in load and 9.3% in extension compared to the 20 kHz small tip contactless mode. The average final extension from the contact mode with the 40 kHz transducer is less than the non-ultrasonic welding results shown in Table 1. This suggests that a high-power ultrasonic vibration level at such a frequency would make the welding less ductile. The 20 kHz transducer outperformed the 40 kHz in all comparable configurations, both in terms of load and extension. The performance difference was particularly notable in contactless setups, where the 20 kHz consistently achieved better results than the 40 kHz, emphasising the efficiency of the lower frequency in this specific welding context. Ductile fracture was observed in every tested sample, where they underwent considerable plastic deformation (necking).

## 4. Ultrasonic Laser Assistance of Aluminium (Al1050) to Copper (Cu101)

**Figure 10:**
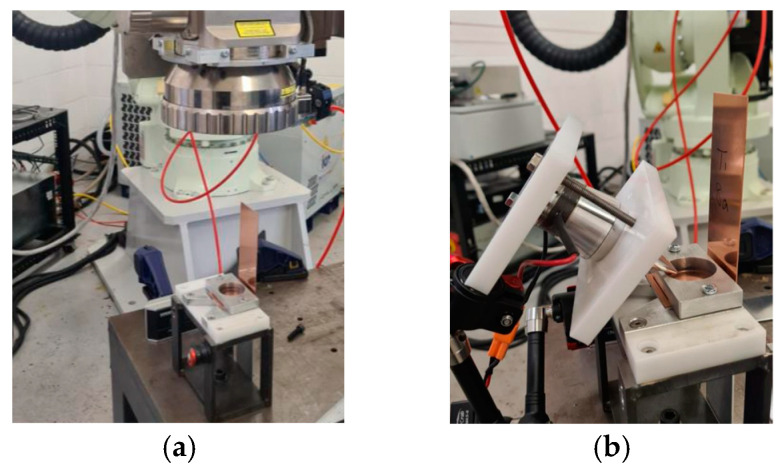
Ultrasonic-vibration-assisted welding of dissimilar materials involves: (**a**) the experimental configuration with the copper (Cu101) plate on top and the aluminium alloy 1050 plate on the bottom, and (**b**) the positioning of the transducer for ultrasonic assistance in welding.

In this section, the HPUTs at 20 kHz and 40 kHz, equipped with small horn tips, operated at 70% power for ultrasonic assistance in laser welding. This investigation explores the use of ultrasonic assistance in the laser welding of dissimilar materials, specifically welding copper (Cu101) and aluminium alloy 1050 plates, as shown in Figure 10a. The method aims to overcome the challenge of forming brittle intermetallic compounds (IMCs) in dissimilar material welds. Both transducers were in contactless mode and positioned at a 45-degree angle towards the weld, as illustrated in Figure 10b. The welding machine used in this section employs the same types of lasers (multi-mode fibre lasers) and utilises identical weld settings and welding fixtures as those described in Section 3.

The same standards and the device, as explained in Section 3.2 and shown in Figure 8, were used to perform the pull tests, and the results are shown in Table 3. This table effectively presents comparative data illustrating the benefits of ultrasonic frequency variations in improving weld strength and ductility. Specifically, the 20 kHz transducer achieved a 45% higher load and a 94% greater extension than the 40 kHz transducer. These results underline the significant advantage of using lower-frequency ultrasonic assistance in enhancing the mechanical properties of the welds, both in terms of strength and ductility.

The tests for ultrasonic laser-assisted welding of the dissimilar materials were repeated, and samples were collected for Scanning Electron Microscopy (SEM) analysis. The inspection was conducted using a Tescan Vega 3 SEM. The analysis focused on identifying features and characteristics of the welds through direct SEM imaging. Microstructure inspection adhered to the ISO 17639:2022 standard [30] and was performed by Loughborough Surface Analysis Limited in Essex, UK.

To reveal the internal structure of the welds, the samples were transversely cut through the centre using a metallurgical abrasive cutting machine, specifically the MetCut 300. Subsequently, to prepare the samples for examination, they were mounted in a 40 mm carbon conductive black resin mount, named CitoPress-15, ensuring secure positioning for accurate observation. For achieving a smooth and polished surface, the Omega-Pol Twin 200 mm manual polishing system was employed. The polishing process involved using sandpaper with grit sizes of 240 and 1200, with the polishing machine set at a speed of 300 rpm. To further refine the surface, colloidal silica and a 3 µm diamond suspension were applied to a polishing cloth with the machine running at 150 rpm.

### 4.1. Baseline Microscopy Results

**Figure 11:**
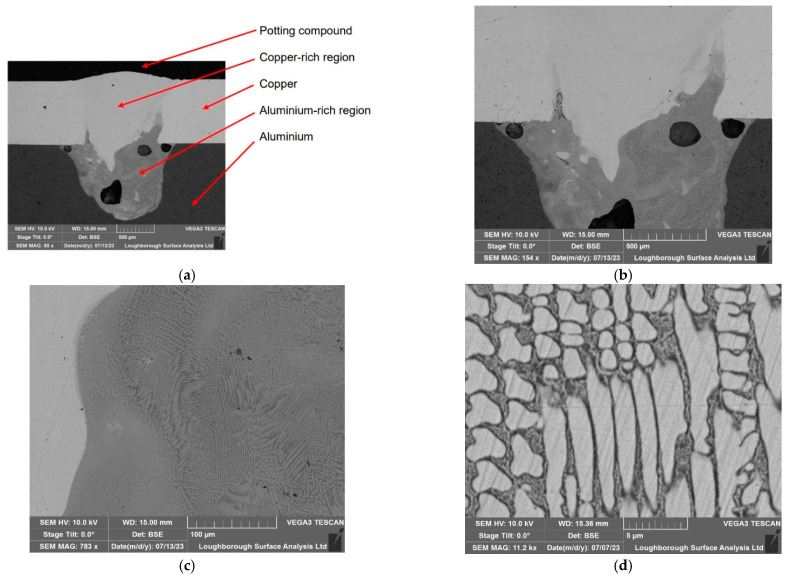
The microscopy results associated with the non-ultrasonic welding: (**a**) 500 µm, explaining the aluminium and copper-rich regions, (**b**) 500 µm, (**c**) 100 µm, and (**d**) 5 µm.

In this section, ultrasonic technology was not employed during the welding process. In the SEM images, different gradients represent variations in the material composition. In this case, copper and aluminium are dissimilar metals which have different atomic numbers, and thus they backscatter electrons at different rates. Metals with a higher atomic number backscatter more electrons than those with a lower atomic number, which is why copper appears brighter in SEM images. Thus, transitions or mixtures between different metals can appear as gradients of colour or brightness. Figure 11 highlights the different intermetallic compounds for welding without sonication. It is important to note that these appear scattered mainly on the copper–aluminium side richer in aluminium due to the higher density of copper.

### 4.2. Ultrasonic-Assisted Welding at 20 kHz

**Figure 12:**
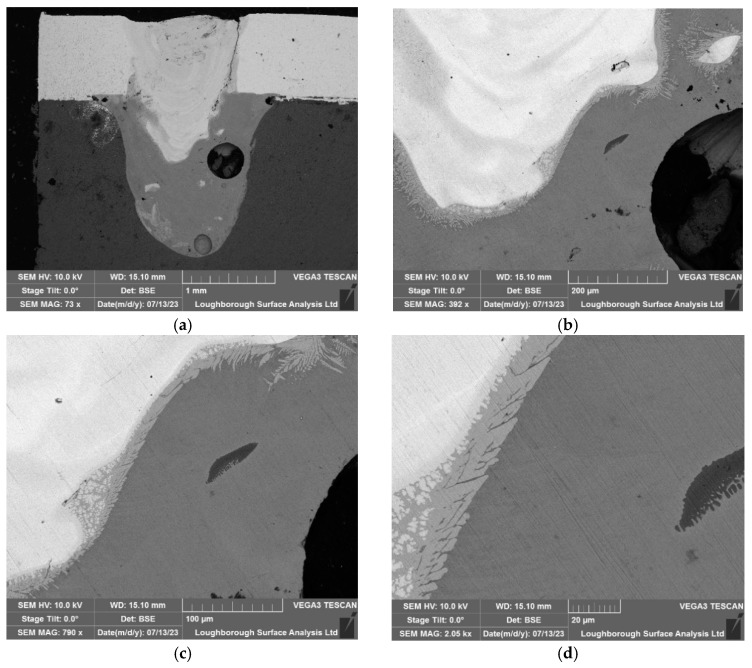
Microscopy of the test associated with the 20 kHz transducer in the contactless mode: (**a**) 1000 µm, (**b**) 200 µm, (**c**) 100 µm, and (**d**) 20 µm.

In this section, a 20 kHz HPUT in contactless mode was used during the laser welding. Figure 12, shows the effect of the ultrasound treatment on the intermetallic mentioned in Section 4.1, where, instead of scattered throughout the copper (Cu101)–aluminium (Al1050) mix, they instead appear mainly in the interface area between both materials. The intermetallic compounds form at the joint interface under all welding conditions, both with and without the use of ultrasonic vibrations. However, as shown in Figure 12, the application of ultrasonic vibrations successfully thins the layer of these intermetallic compounds at the interface, which in turn enhances the mechanical properties of the joints [31,32].

### 4.3. Ultrasonic-Assisted Welding at 40 kHz

**Figure 13:**
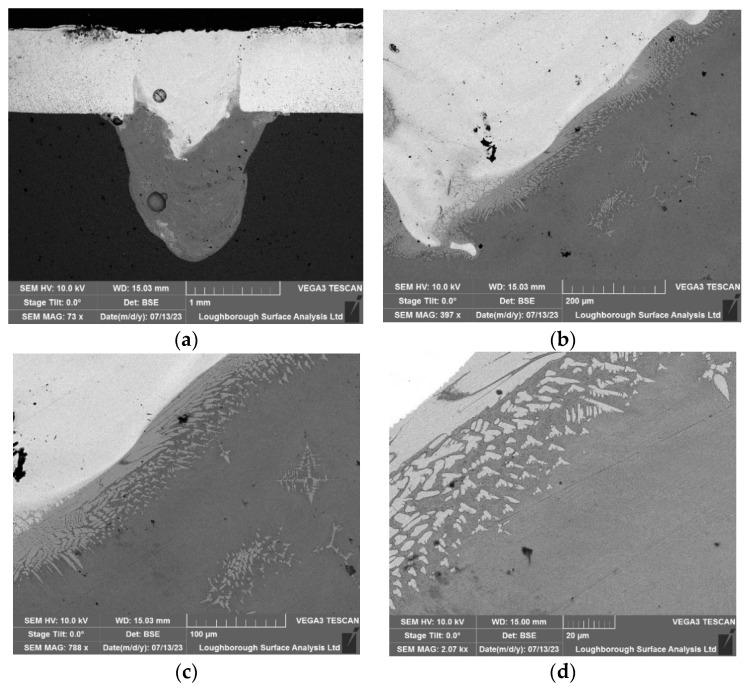
Microscopy of the test associated with the 40 kHz transducer in the contactless mode: (**a**) 1000 µm, (**b**) 200 µm, (**c**) 100 µm, and (**d**) 20 µm.

In this section, the laser welding process involved ultrasonic assistance from a 40 kHz HPUT. As shown in Figure 13, the ultrasound treatment with a 40 kHz HPUT influences the intermetallic compounds, preventing their dispersion throughout the mixture of the two metals. Ultrasonic assistance, as shown in Figure 13, effectively controls the placement and reduces the spread of intermetallic compounds compared to the non-assisted method shown in Figure 11. The microstructural improvements evident in Figure 13, achieved through ultrasonic assistance, correlate with enhanced mechanical properties, such as increased tensile strength and ductility, as detailed in Table 3.

## 5. Numerical Simulation of Vibration Transmission to the Molten Pool Area

This section aims to provide an estimation regarding the pressure transmission into the molten pool area, with the configurations illustrated in Figure 2.

**Figure 14:**
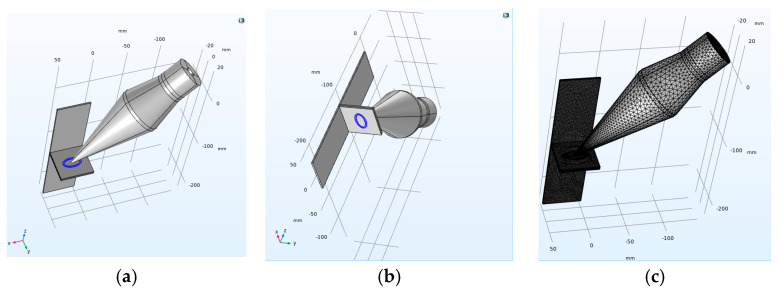
The model configuration and the location of the applied vibration: (**a**) the highlighted area is considered molten copper; (**b**) the same location is considered molten aluminium; and (**c**) the meshed model.

**Figure 15:**
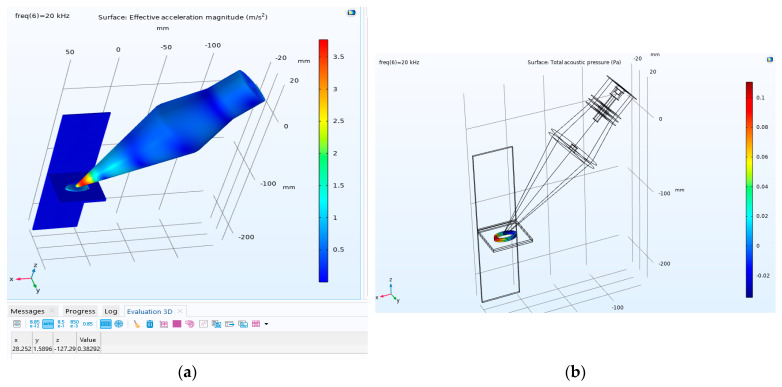
Applying sonication to the molten pool area at 20 kHz. (**a**) Acceleration amplitude: the table below displays the acceleration amplitude values measured at the center of an oval and (**b**) the acoustic pressure in the molten pool area.

To simplify the model, it is assumed that the tip of the transducer is in contact with the centre of the oval-shaped weld, as shown in Figure 14. The top oval shape highlighted in Figure 15a is assumed to be molten copper (Cu101), which melts at approximately 1080 °C, while the bottom one is aluminium (Al1050), which becomes molten at 660 °C.

The model integrates pressure acoustics, solid mechanics, and electrostatics, with relevant equations assigned to each respective medium. In the solid mechanics domain, reflective boundaries are applied at the area of the welding molten pool. A comprehensive thermal simulation was conducted by Dimopoulos et al. [33]; however, in this simulation, only the effect of heating on vibration transmission to the molten pool area is considered. In the domain of electrostatics, the behaviour of piezoelectric ceramics is governed solely by the equations of solid mechanics. Free tetrahedral elements are employed, with the maximum mesh size considered to be one-sixth of the wavelength [34]. In this study, the piezoelectric materials modelled were glass and zirconate titanate (PZT-4). All material properties were selected from the material library of COMSOL Multiphysics. The main body of the transducer is assumed to be made of steel, and aluminium material is selected for the horn. The material properties used in this simulation are given in Table 4.

The acceleration value shown in Figure 15a is similar to the one shown in Figure 5. The obtained value shown in Figure 15b indicates that an acoustic pressure of approximately 0.1 Pa appears in the molten pool area. According to the theoretical results by Teyeb et al. [20], specifically Figure 4 in that paper, no ultrasound cavitation will develop in the molten pool area as the threshold for the cavitation at a frequency of 20 kHz is approximately $10^{5}$ Pa.

## 6. Conclusions

The study illustrates the effectiveness of integrating ultrasonic vibration in laser welding processes for EV battery connectors. Employing both 20 kHz and 40 kHz High-Power Ultrasound Transducers (HPUTs) in contact and contactless modes, enhancements were observed in the welding strength and microstructural integrity of the welds.

Experimental results highlighted that the 20 kHz transducer in contactless mode demonstrated superior performance, showing a 10% increase in load and 27% increase in extension compared to welding without ultrasonic assistance. On the other hand, the 40 kHz transducer, while still improved over non-ultrasonic methods, showed less pronounced benefits. The best performance with 40 kHz (small tip contactless) fell short by approximately 6.4% in load and 9.3% in extension compared to the 20 kHz small tip contactless mode. This suggests that lower-frequency ultrasonic assistance (20 kHz) is more effective in this specific context.

Microstructural analysis through microscopy substantiated that ultrasonic assistance aids in reorganising the intermetallic position in such a fashion that leads to increasing the ductility and strength of the weld. The 40 kHz transducer, despite its slightly lower performance in mechanical testing, still positively impacted the microstructural characteristics of the welds, demonstrating reduced homogenisation of copper (Cu101) into aluminium (Al1050), which aligns with the expected influence of ultrasonic vibrations on material behaviour during welding. In conclusion, while both 20 kHz and 40 kHz transducers enhance the welding quality, the 20 kHz frequency is more efficient in this context.

The numerical simulation reveals that the vibration transmission into the molten pool area is well below the cavitation initiation threshold, and all improvements to the mechanical properties of the weld are related to the ultrasonic waves.

## Figures and Tables

**Table 1:** Pull test results associated with the 20 kHz HPUT with small and large horn tips, alongside laser welding without ultrasonic assistance. All tests were conducted in triplicate.

Horn Type/Diameter (mm)	Excitation Mode/Angle	Average Load (N)	Average Final Extension (mm)
Small tip/3.3	Contactless/45°	561.0	14.0
Large tip/33	Contact/0°	530.7	10.7
Large tip/33	Contactless/45°	533.1	13.2
No ultrasonic assistance	N/A	509.0	11.0

**Table 2:** Pull test results associated with the 40 kHz HPUT with small and large horn end tips, repeated three times.

Horn Type/Diameter (mm)	Excitation Mode/Angle	Average Load (N)	Average Final Extension (mm)
Small Tip/2.25	Contactless/45°	527.0	12.7
Large Tip/22.5	Contact/0°	516.0	9.7
Large Tip/22.5	Contactless/45°	520.4	13.0
No ultrasonic assistance	N/A	509.0	11.0

**Table 3:** Pull test results associated with the 20 kHz and 40 kHz HPUTs with the small horn end tip (focused horns). All the tests were repeated three times.

Frequency	Excitation Mode/Angle	Average Load (N)	Average Final Extension (mm)
20 kHz	Contactless/45°	246.8	20.8
40 kHz	Contactless/45°	170.1	10.7
No ultrasonic assistance	N/A	110.0	4.8

**Table 4:** Material properties used in the simulation.

Properties	Young’s Modulus (Pa)	Density (kg/m³)	Loss Factor	Wave Speed (m/s)
Aluminium (Al1050)	$7\;\times \;10^{9}$	2700	0.003	5120
Copper (Cu101)	$110\;\times \;10^{9}$	8960	0.005	3810
Steel	$203\;\times \;10^{9}$	7850	0.0006	5950
Lead Zirconate Titante	$245\;\times \;10^{9}$	7600	0.02	-
Molten aluminium (Al1050)	-	2375	-	-
Molten copper (Cu101)	-	8020	-	-

## Data Availability

Data are contained within the article.
